# Supplementary material for: Modulation of Hepatic Functions by Chicory (Cichorium intybus L.) Extract: Preclinical Study in Rats
Source: Pharmaceuticals (Basel). 2023 Oct 16;16(10):1471. doi: 10.3390/ph16101471 (PMC10609820; doi:10.3390/ph16101471)
Supplement: Supplementary file 1 [file pharmaceuticals-16-01471-s001.zip › pharmaceuticals-2452707-supplementary.pdf]

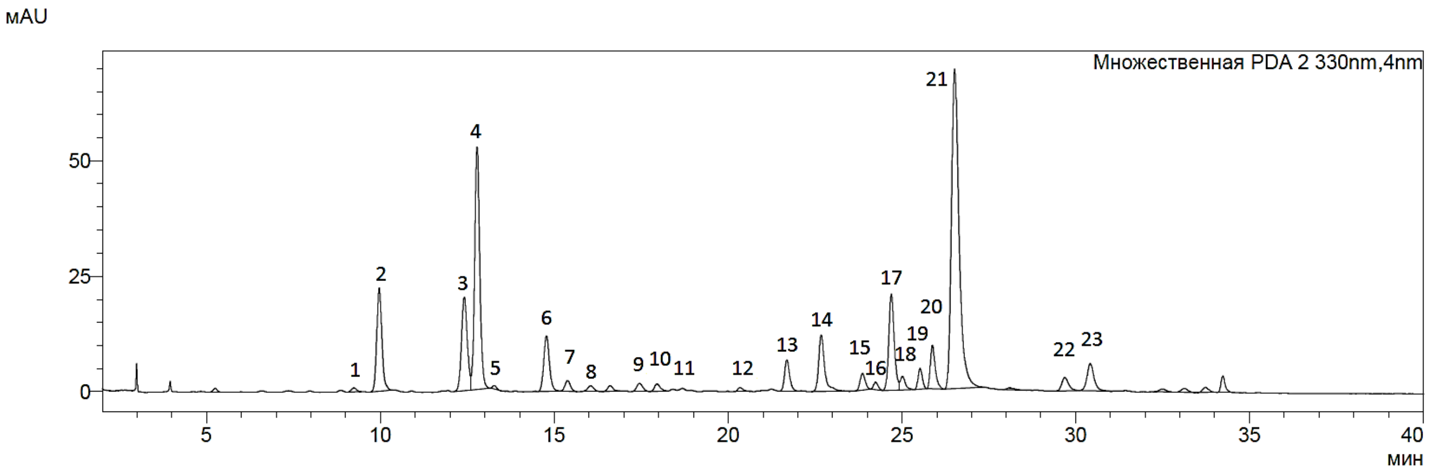

Figure S1. HPLC chromatogram of polyphenolic compounds of chicory extract (330 nm).

Table S1. Phenolic compounds identified in CE.

Compound	Compound number (see fig 1S)	Retention time, min	Molecular weight, Da	Positive ionization, m/z, fragments	Negative ionization, m/z, fragments
Dihydroxybenzoic acid	1	9.241	154	172 [M+H_2O]$^-$	153 [M-H]$^-$
Cichoriin	2	9.969	340	179 (esculetin)<br>341 [M+H]$^-$<br>363 [M+Na]$^-$<br>681 2M+H<br>703 [2M+Na]$^-$	177 (esculetin)<br>339 [M-H]$^-$<br>679 [2M-H]$^-$
Caftaric acid	3	12.414	312	313 [M+H]$^-$<br>330 [M+H_2O]$^-$<br>335 [M+Na]$^-$	149 (tartaric acid)<br>179 (caffeic acid)<br>311 [M-H]$^-$<br>357 [M+HCOOH]$^-$
Chlorogenic acid	4	12.783	354	163 caffeic acid – H_2O)<br>355 [M+H]$^-$<br>377 [M+Na]$^-$<br>731 [2M+Na]$^-$	191 (quinic acid)<br>353 [M-H]$^-$<br>707 [2M-H]$^-$
n- hydroxybenzoic acid	5	13.270	138	139 [M+H]$^-$	137 [M-H]$^-$

Esculetin	6	14.778	178	179 [M+H]$^-$<br>201 [M+Na]$^-$<br>379 [2M+Na]$^-$	177 [M-H]$^-$<br>355 [2M-H]$^-$
Caffeic acid	7	15.383	180	163 [M-H_2O]$^-$<br>181 [M+H]$^-$<br>198 [M+H_2O]$^-$	179 [M-H]$^-$
Coumaroylquinic acid	8	16.612	338	147 (quinic acid)<br>339<br>361 [M+Na]$^-$<br>699 [2M+H]$^-$	191 (quinic acid)<br>337<br>675 [2M-H]$^-$
Coutaric acid (coumaroiltartaric)	9	17.49	296	147 (coumaric acid)<br>297 [M+H]$^-$<br>314 [M+H_2O]$^-$<br>319 [2M+Na]$^-$	295 [M-H]$^-$
Feruloylquinic acid	10	17.955	368	177 (ferulic acid - H_2O)<br>195 (ferulic acid)<br>369 [M+H]$^-$<br>391 [M+Na]$^-$<br>759 [2M+Na]$^-$	367 [M-H]$^-$<br>735 [2M-H]$^-$
Fertaric acid (Feruloyltartaric *acid*)	11	18.561	326	195 (ferulic acid)<br>327 [M+H]$^-$<br>344 [M+H_2O]$^-$<br>349 [M+Na]$^-$	325 [M-H]$^-$
Rutin	12	20.344	610	303 (quercetin)<br>611 [M+H]$^-$<br>633 [M+Na]$^-$	609 [M-H]$^-$
Isoquercetin	13	21.693	464	303 (quercetin)<br>465 [M+H]$^-$<br>487 [M+Na]$^-$<br>951 [2M+Na]$^-$	463 [M-H]$^-$<br>927 [2M-H]$^-$

Quercetin glucuronide	14	22.680	478	303<br>479 $[M+H]^-$<br>501 $[M+Na]^-$	477 $[M-H]^-$<br>955 $[2M-H]^-$
Isochlorogenic acid B (dicofeoylquinic)	15	23.868	516	499 $[M-H_2O]^-$<br>517	515<br>561 $[M+Na]^-$
Astragalin	16	24.244	448	287 (kaempferol)<br>449 $[M+H]^-$<br>471 $[M+Na]^-$<br>919 $[2M+Na]^-$	447 $[M-H]^-$<br>895 $[2M-H]^-$
Isochlorogenic acid A (dicofeoylquinic)	17	24.697	516	163 (caffeic acid H_2O)<br>181 (caffeic acid)<br>499 $[M-H_2O]^-$<br>517<br>539 $[M+Na]^-$	353 (caffeine-quinine acid)<br>515<br>561 $[M+Na]^-$
Kaempferol/ luteolin glucuronide	18	25.018	462	287 (aglycone)<br>463 $[M+H]^-$<br>485 $[M+Na]^-$	461 $[M-H]^-$<br>923 $[2M-H]^-$
Isorhamnetin glucuronide	19	25.524	492	317 (isoramnetin)<br>493 $[M+H]^-$<br>515 $[M+Na]^-$	491 $[M-H]^-$
Isochlorogenic acid C (dicofeoylquinic)	20	25.878	516	163 (caffeic acid – H_2O)<br>181 (caffeic acid)<br>499 $[M-H_2O]^-$<br>517<br>539 $[M+Na]^-$	515
Chicoric acid	21	26.519	474	163 (caffeic acid - H_2O)<br>181 (caffeic acid)<br>475 $[M+H]^-$<br>492 $[M+H_2O]^-$<br>497 $[M+Na]^-$<br>971 $[2M+Na]^-$	293 [M- caffeic acid $-H_2O]^-$<br>311 [M- caffeic acid$]^-$<br>473 $[M-H]^-$<br>947 $[2M-H]^-$

Luteolin	22	29.690	286	287 $[M+H]^-$<br>309 $[M+Na]^-$	285 $[M-H]^-$
Coumaroyl-feruloyltartaric acid	23	30..416	488	177 (феруловая кислота - H_2O)<br>195 (ferulic acid a)<br>327 (feruloyltartaric acid)<br>489<br>506 $[M+H_2O]^-$<br>511 $[M+Na]^-$	325 (feruloyltartaric acid)<br>487
